# Health care: the challenge to deal with uncertainty and value judgment

**DOI:** 10.1186/s12962-015-0035-y

**Published:** 2015-05-01

**Authors:** Marcos Bosi Ferraz

**Affiliations:** Department of Medicine, Escola Paulista e Medicina, Federal University of São Paulo, São Paulo, Brazil; São Paulo Center for Health Economics, Escola Paulista de Medicina, Federal University of São Paulo, São Paulo, Brazil; GRIDES, Federal University of São Paulo, Rua Botucatu, 740, São Paulo, 04023-062 Brazil

## Abstract

The exponential increase of knowledge in the life sciences field, more specifically in health sciences, in the past few years has brought additional levels of complexity when deciding and implementing strategies in the health care system. A predominantly paternalistic way to decide about available options to maintain or improve individual or collective health has been moving to a shared-decision model considering the empowered patient. In spite of the reduction of uncertainty when making health and health care decisions due to the advancement in scientific methods, and, in spite of the asymmetry of information, knowledge and power to make decisions, we are progressively recognizing the importance of individuals, the target of the intervention, to express their preferences and to take an active role in the decision making process. Health care stakeholders, recognizing the scarcity of resources available and the fortunate ever increasing amount of applicable knowledge and its corresponding interventions to improve the population quantity and quality of life, should stimulate society to address and discuss health care issues that will guide critical choices and define health care priorities based mostly on judgment and the best evidence available.

## Commentary

The exponential increase of knowledge in the life sciences field, more specifically in health sciences, in the past few years has brought additional levels of complexity when deciding and implementing strategies in the health care system. In the past decades we have been observing a desperate and obsessive investment in the advancement of rigorous scientific methods to better allow us understand the phenomena under study or observation. The main aim has been to decrease the uncertainty of the phenomena under study, or in other words, to allow it to be as close as possible to certainty. Since health care decisions deal with human life, we want to be as certain as possible in order to promote health, and to prevent or alleviate suffering.

Concurrently, a predominantly paternalistic way to decide about available options to maintain or improve individual or collective health has been moving to a shared-decision model considering the empowered patient [1-3]. In spite of the reduction of uncertainty when making health and health care decisions due to the advancement in scientific methods, and, in spite of the asymmetry of information, knowledge and power to make decisions, we are progressively recognizing the importance of individuals, the target of the intervention, to express their preferences and to take an active role in the decision making process. Due to the uncertainty and/or different perceptions of health or intervention risks as well as distinct set of values and preferences, individuals have been called to contribute with decisions regarding their own health.

The construct of health care empowerment is defined as the process and state of being engaged, informed, collaborative, committed, and tolerant of uncertainty regarding health care [4]. Although empowerment may place greater demands on health care professionals, it ideally should not be viewed as an intrusion into the decision making process. It is also important to point out that individuals have the potential to contribute to a common goal within a colective process of social change [5].

**Figure 1:**
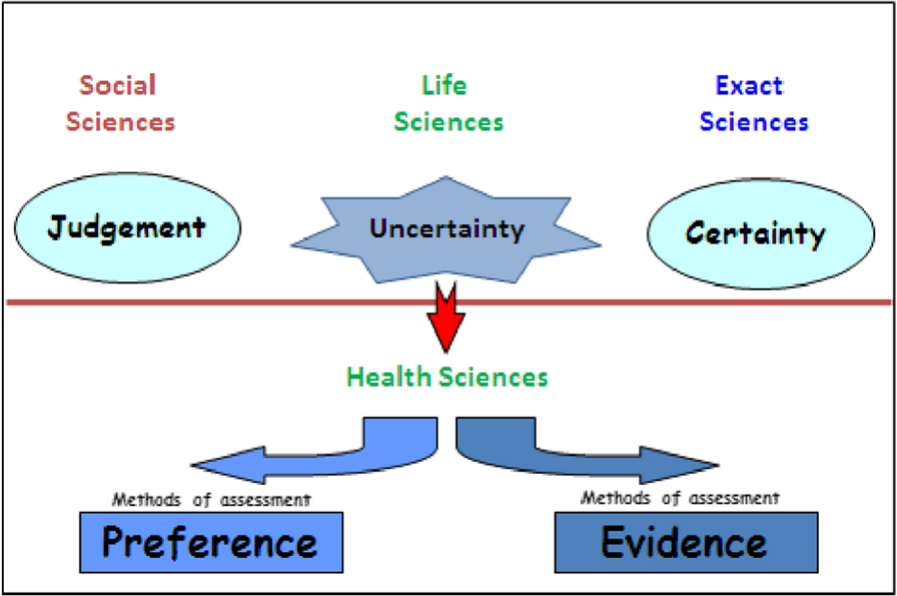
Current trends of Health Sciences.

Figure 1 schematically presents trends in health sciences. Society and health care systems face the key challenge of, simultaneously, decreasing the level of uncertainty and increasingly respecting the individual and societal value judgment in the health care decision making process.

In this process the individual decision affects the collective and the collective decision imposes restrictions on individuals. The definition and knowledge of the restrictions should ideally be defined beforehand, be well known by all individuals and it should follow equity principles. The respect for individual and collective preferences, well discussed and defined, and the moral and ethical values expressed by our society should, therefore, guide all decisions [6].

It is also important to point out that health care, besides being highly complex, it is dynamic and very creative. It “sells” hope, beyond health, in an environment of different parties, interests and incentives, which sometimes are perverse. Health care system in most countries (being public, private or mixed) is based on insurance, which means that population shares the financial risk and, from time to time, the collective agreement has to be renewed. One of the challenges it faces is to clearly define its objectives for the short-, medium- and long-term in a constantly changing environment. The definition of its objectives should be based on the burden of diseases, the continuing critical appraisal of the evolving scientific evidence, the recognition of the disposable infra-structure (including health care professionals), and the understanding of individual and societal set of values and preferences; the acceptance of the finite nature of financial resources available for a specific period of time is crucial to allow a mature and responsible decision-making process. Due to the scarcity of resources health care priorities should be defined.

Currently most health care systems operate as hope-driven systems, which are very much aligned with supply-driven systems that interfere with the priority setting process. Health care systems, however, should be anchored and powered by priority setting agreements that favor the fulfillment of health care individual and societal needs. The hope-driven system should, therefore, be gradually transformed to a value-driven or need-based system.

In this scenario, healthcare systems in many developing countries, like Brazil, currently face additional challenges: how to meet the demand for 21st century standards of health care and technology with funds that, as a percentage of the gross domestic product, remain lower than what developed nations were investing in health in the 1980s. And, furthermore, how can developing countries meet such expectations when they are still dealing with health problems that rich countries had overcome 40 or 50 years ago? [7]. Considering the ever increasing demand for health care services and products and the perception of progressively and relatively increasing scarcity of resources, health care decisions should be even more based and justified by the best evidence available and individual and societal preferences.

Due to scarcity of resources, tough and transparent health care decisions that affect society have to be made anyway. Taking breast cancer just as an example, the trade-offs between investing in prevention strategies to avoid new cases or even to identify cases and to delay the progression of the disease already established have to be balanced with the opportunity cost of treating the diagnosed cases. The same trade-off may arise when discussing the appropriateness of the health care investment for the young and the very old people. In both examples, certainly there are plenty of evidence-based options that may justify health care decisions. The opportunity cost of the investments in these examples goes beyond the limits of health sciences. Society should be prepared to discuss the ethical and distributive justice concepts that this scenario presents [8,9]. Many economic tools have been developed and used to inform decisions, but, sometimes, we are distracted by the passion with some sophisticated methods and forget the real world health care system problems and choices are much simpler than we expect. Health economics should note be used as “l′art pour l′art”. The excess use of methods and tools without fully defining the basic gols and philosophical principles of the health care system and without evaluating the fitness of these measures to reaching these goals may not contribute to na efficient improvement of population health [8].

The economic evaluation of health care programs, in general, has difficulty accomodating the concept of rights. It proposes triage as the model of a rational delivery system, with no place for traditional ethical limits and obligations [10].

The equity versus efficiency dilemma has been virtually ignored in the political debate, ofte leading to inconsistent judgements in the development of health policies [11].

There is an urgent need to discuss what society judges the more appropriate decision based on its values and the aligned doctrine of its health care system.

In conclusion, in this increasingly complex environment, health sciences have to continuously and progressively develop methods and tools to decrease the uncertainty. Individuals should be progressively educated and empowered to take part in the decisions involving its own health. Providers should focus on creating the bridges to help individuals to understand the probabilities, risks and chances, values and preferences of alternative available options, and stimulate them to take their role in the decision process [12,13]. Elected representatives, policy makers and managers, recognizing the scarcity of resources available and the fortunate ever increasing amount of applicable knowledge and its corresponding interventions to improve the population quantity and quality of life, should stimulate society to address and discuss health care issues that will guide critical choices and define health care priorities based mostly on judgment and the best evidence available.
